# The Immunological and Virological Consequences of Planned Treatment Interruptions in Children with HIV Infection

**DOI:** 10.1371/journal.pone.0076582

**Published:** 2013-10-23

**Authors:** Nigel Klein, Delali Sefe, Ilaria Mosconi, Marisa Zanchetta, Hannah Castro, Marianne Jacobsen, Hannah Jones, Stefania Bernardi, Deenan Pillay, Carlo Giaquinto, A. Sarah Walker, Diana M. Gibb, Anita De Rossi, on Behalf of the Paediatric European Network for Treatment of AIDS (PENTA) 11 Trial Team

**Affiliations:** 1 Institute of Child Health, University College London, London, United Kingdom; 2 AIDS Reference Center, University of Padova, Padova, Italy; 3 Medical Research Council Clinical Trials Unit, London, United Kingdom; 4 Ospedale del Bambino Gesù, Rome, Italy; 5 University College London/Medical Research Council Centre for Medical Molecular Virology, University College London Medical School, London, United Kingdom; 6 Department of Paediatrics, University of Padova, Padova, Italy; New York University, United States of America

## Abstract

**Objectives:**

To evaluate the immunological and viral consequences of planned treatment interruptions (PTI) in children with HIV.

**Design:**

This was an immunological and virological sub-study of the Paediatric European Network for Treatment of AIDS (PENTA) 11 trial, which compared CD4-guided PTI of antiretroviral therapy (ART) with continuous therapy (CT) in children.

**Methods:**

HIV-1 RNA and lymphocyte subsets, including CD4 and CD8 cells, were quantified on fresh samples collected during the study; CD45RA, CD45RO and CD31 subpopulations were evaluated in some centres. For 36 (18 PTI, 18 CT) children, immunophenotyping was performed and cell-associated HIV-1 DNA analysed on stored samples to 48 weeks.

**Results:**

In the PTI group, CD4 cell count fell rapidly in the first 12 weeks off ART, with decreases in both naïve and memory cells. However, the proportion of CD4 cells expressing CD45RA and CD45RO remained constant in both groups. The increase in CD8 cells in the first 12 weeks off ART in the PTI group was predominantly due to increases in RO-expressing cells. PTI was associated with a rapid and sustained increase in CD4 cells expressing Ki67 and HLA-DR, and increased levels of HIV-1 DNA.

**Conclusions:**

PTI in children is associated with rapid changes in CD4 and CD8 cells, likely due to increased cell turnover and immune activation. However, children off treatment may be able to maintain stable levels of naïve CD4 cells, at least in proportion to the memory cell pool, which may in part explain the observed excellent CD4 cell recovery with re-introduction of ART.

## Introduction

The Paediatric European Network for Treatment of AIDS (PENTA) 11 trial was a pilot study comparing CD4-guided planned treatment interruptions (PTI) of antiretroviral therapy (ART) with continuous therapy (CT) in HIV-1-infected children who had achieved long-term plasma HIV-1 RNA suppression and substantial immune reconstitution. The key findings were that no serious clinical outcomes occurred in children undergoing PTIs, [1] and that following re-introduction of ART, the PTI group recovered CD4 and re-suppressed viral load to the same extent as the CT group [2].

The results of studying a range of structured treatment interruption strategies in Adults infected with HIV have generally been disappointing [3]–[7]. Patients exhibit lower CD4 counts, experience more infections, have higher mortality rates, and are at greater risk of virological failure upon ART reinstitution. Although PENTA 11 was only a small pilot study, there are reasons why children may be able to better tolerate or even benefit from the PTI. Firstly, the recovery rates of naïve CD4 T cells during ART are significantly higher for children under 3 years of age than for older children, and ten to forty times higher than for adults, [8], [9] most likely because children have a higher thymic output than adults [10]. Secondly, largely as a result of immunological immaturity, infants have higher rates of viral replication than older children and adults [11]. However if virus can be successfully suppressed with ART during early childhood, better viral control may be achieved in the context of both a more mature immune system and good thymic output, if, or when, therapy is interrupted [12].

Thus interrupting therapy, and re-exposing children to HIV, at a point where there is a large population of naive T cells and the immune system is relatively more mature than at primary (vertical) infection may have different consequences from adults. We therefore performed in-depth immunological and virological analyses in a subset of children randomised to CT and PTI to address this question.

## Methods

PENTA 11 was an open, multicentre, randomised, phase II, trial (ISRCTN36694210) in HIV-1 infected children aged 2–15 years, on any ART regimen containing three or more drugs which they had taken for ≥24 weeks. Eligibility criteria included the two most recent plasma HIV-1 RNA to be <50 copies/ml, (screening and prescreening), and CD4% to be ≥30% (ages 2–6 years) or ≥25% and CD4 count ≥500 cells/mm^3^ (7–15 years) [1]. Children were randomised to either CT or to an open strategy of CD4-guided PTI. ART was restarted if confirmed CD4% was less than 20% or more than 48 weeks had been spent off ART. A substudy to examine the influence of PTI on immunological and virological parameters was performed on stored samples in a subset of children.

HIV-1 RNA and CD4 and CD8 lymphocyte subsets were quantified locally on fresh samples collected at 0, 2, 4, 8, 12 weeks after enrolment and then 12-weekly. In some centres, CD45RA and CD45RO subpopulations of CD4 and CD8 cells were also evaluated on fresh samples (including for the 36 children in the substudy described below). In centres able to separate and store cells, an additional 10 ml of whole blood was collected in EDTA at each visit; peripheral blood mononuclear cells (PBMC) were isolated by density gradient centrifugation, divided into aliquots, and frozen.

### Immunophenotyping

In 20 UK children (11CT, 9PTI) and 12 Italian children (5CT, 7PTI), aliquots were thawed and cell suspensions incubated with antibodies: anti-CD3 [fluorescein isothiocyanate (FITC)], anti-CD4 [peridinin chlorophyll protein (PerCP)], anti-CD8 (PerCP), anti-CD38 [phyoerythrin (PE)], anti-CD45RA [allophycocyanin (APC)], and anti-CD31 (PE), all from Becton-Dickinson (Becton-Dickinson Biosciences Pharmingen, San Diego, CA, USA). Appropriate isotypic controls (mouse IgG1-PE and IgG2b-APC) were used to evaluate non-specific staining. Cells were then washed with Automacs Buffer (Milteny Biotec Inc. Auburn, CA, USA) and resuspended in PBS supplemented with 1% paraformaldehyde. All samples were analyzed by four-color flow cytometry using a FACS Calibur (Becton-Dickinson) with a 488 nm argon-ion laser and a 635 nm red diode laser. 50,000 events were collected in the lymphocyte gate using morphological parameters (Forward and Side scatter). Data were processed using CellQuest Pro Software (Becton-Dickinson) and analyzed using the Summit Software version 4.3 (Dako, Glostrup, Denmark).

### Viral load quantification

In 16 Italian children (7CT, 9PTI) (12 with immunophenotyping plus 4 others with insufficient samples for immunophenotyping), cell-associated HIV-1 DNA was measured in PBMC by real time PCR, as previously described [13]. The HIV-1 copy number was normalized against β-actin copies, and final results expressed as HIV-1 DNA copies/10^6^ PBMC.

### Quantification of lipopolysaccharide

In the same 16 children with cell-associated HIV-1 DNA measured, lipopolysaccharide (LPS) was determined in plasma samples diluted five-fold with endotoxin-free water and then heated to 70°C for 10 min to inactivate plasma proteins. Plasma LPS was quantified using a chromogenic assay (Limolus Amebocyte Lysate QCL-1000), as previously described [14].

### Statistical analysis

Baseline characteristics of children in the substudy were compared by randomised group, and children in the main study were compared to those in the substudy, using chi-squared test for categorical variables and the t-test for continuous variables. Changes from baseline in laboratory measurements were estimated using normal regression of actual measurements adjusting for baseline. Generalised estimating equations were used for global tests of differences between randomised groups over 48 weeks, adjusted for baseline [15]. Relative changes from baseline in HIV-1 DNA were estimated using normal regression of log_10_ transformed measurements. Unavailable data due to non-attendance, failure to collect the sample, or failure to obtain satisfactory data upon thawing of the frozen PBMCs were treated as missing at random. In the PTI group, measurements taken after restarting ART were excluded; see [1] for details of children restarting ART during the study. All analysis used Stata statistical software, version 10.1 (StataCorp., College Station, Texas, USA).

### Role of funding source

Representatives of the sponsor, the PENTA Foundation, were part of the study team and therefore were involved in study design, coordination, data collection, data analysis, data interpretation, and writing of the report. The corresponding author had full access to all the data in the study and had final responsibility for the decision to submit for publication.

### Ethics

The protocol for this study was approved by the ethics committee for each participating centre. Patients included in this sub study were recruited from the UK, approval from Trent MREC, and from Italy, approval from Comitato Etico per la Sperimentazione, Azienda Ospedaliera di Padova. All parents/guardians gave written consent, and children gave written assent, according to their age and knowledge of HIV status.

## Results

109 children (53CT, 56PTI) were included in the main PENTA 11 analysis, randomised between November 2004 and December 2006. At baseline the median age was 9 (range 2–16) years, median CD4% 37% (interquartile range (IQR): 33,41) and CD4 990 (763,1248) cells/mm^3^. No child died or had a new CDC C diagnosis. One child (2%) in CT versus four (7%) in PTI (difference +5%, 95% confidence interval (CI) −2% to +13%; p = 0.2) reached a CD4 outcome (3PTI CD4% <15% (aged <7 years), 1CT 1PTI CD4% <15% and CD4 count <200 cells/mm^3^ (aged ≥7 years) [1]. Of 56 children randomized to PTI, 19 reached the CD4-guided ART restart criteria between 6 and 42 weeks after stopping ART, 32 restarted ART only because they had been off ART for 48 weeks, and four restarted for other reasons.

Baseline characteristics of the 36 children in the substudy (median age 9 (range 3–16) years, median CD4% 37% (IQR: 33,42) and CD4 count 990 (835,1276) cells/mm^3^) were similar to all children in the study, although CD8% was slightly lower (Table 1). Similarly, there were no differences in baseline characteristics between the CT and PTI children within the substudy (Table 1). Of 18 children in the PTI substudy group, 4 reached the CD4-guided ART restart criteria 8, 12, 19 and 37 weeks after stopping ART, 12 restarted ART only because they had been off ART for 48 weeks and two restarted for other reasons at weeks 16 and 87.

**Table 1:** Baseline Characteristics.

	Main trial		Substudy		Substudy	Overall
	CT	PTI	CT	PTI	p-value	p-value
	(n = 53)	(n = 56)	(n = 18)	(n = 18)	CT vs PTI	main trial vs substudy
Male (%)	22 (42%)	27 (48%)	9 (50%)	10 (56%)	0.7	0.4
Age (years)						
median [IQR]	9.9 [6.4,12.3]	9.0 [6.7,11.9]	9.9 [5.8,11.6]	9.0 [6.0,10.9]	1.0	0.6
≥2 years to <7 years	15 (28%)	17 (30%)	6 (33%)	7 (39%)		
≥7 years to <11 years	18 (34%)	22 (40%)	8 (44%)	8 (44%)	0.9	0.3
≥11 years to <16 years	20 (38%)	17 (31%)	4 (22%)	3 (17%)		
CDC disease stage						
% N/A/B/C	17/23/26/34	20/34/28/18	1/4/7/6	3/4/8/3	0.6	0.4
HIV-1 RNA:						
≥50 copies/ml at baseline^a^	3 (6%)	8 (14%)	2 (11%)	3 (17%)	0.6	0.5
CD4 parameters, median [IQR]						
CD4% of total lymphocytes	37 [34,40]	37 [33,42]	35 [33], [38]	37 [33,44]	0.1	1.0
CD4 (cells/mm^3^)	965 [741,1222]	967 [844,1302]	999 [786,1151]	989 [860,1321]	0.4	0.9
CD8 parameters, median [IQR]						
CD8% of total lymphocytes	31 [27], [34]	33 [26], [37]	29 [25], [32]	25 [23], [33]	0.3	0.02
CD8 (cells/mm^3^)	790 [592,1020]	871 [615,1070]	765 [540,1010]	643 [544,961]	0.5	0.2

a All children had HIV-1 RNA <50 copies/ml at screening and pre-screening. HIV-1 RNA was assumed to be at least 50 copies/ml at baseline for 11 children [six children (two CT and four PTI) <100 copies/ml, two children (one CT and one PTI) <200 copies/ml and three children (PTI) > = 200 copies/ml: range 240–2430 copies/ml. Note: chi-squared test and t-test were used to compare categorical and continuous characteristics respectively.

CT  =  continuous therapy; PTI  =  planned treatment interruption; IQR  =  interquartile range; CDC  =  Centers for Disease Control and Prevention.

### Changes in HIV-1 RNA, CD4 and CD8 cells

**Figure 1:**
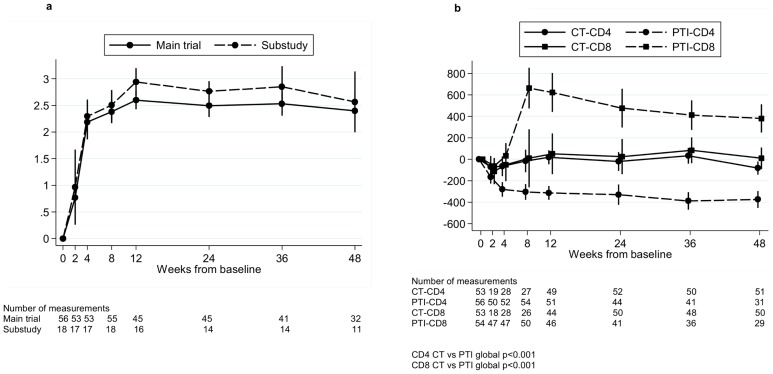
Change in HIV-1 RNA, CD4 and CD8 cells from baseline in the PTI group. Change in HIV-1 RNA from baseline for all children in the trial in the PTI (planned treatment interruption) group off ART (antiretroviral therapy) and children in the substudy in the PTI group off ART (a). Change in CD4 and CD8 from baseline for all children in the trial in the PTI (planned treatment interruption) group off ART (antiretroviral therapy) and in the CT (continuous ART) group (b). Changes from baseline were estimated using normal regression of actual measurements adjusting for baseline using the overall mean at randomisation as the reference category.

As previously described [1], HIV-1 RNA increased rapidly in the PTI group over the first 4 weeks off ART, reaching a peak at ∼12 weeks (mean (95% CI) change 0–12 weeks 2.6 (2.4,2.8) log_10_ copies/ml) and remaining constant to 48 weeks (Figure 1a). CD4 cell count also fell sharply in the PTI group (mean (95% CI) change 0–12 weeks −312 (−377,−248) cells/mm^3^), and then stabilized (Figure 1b). CD8 cell count increased to reach a peak at eight weeks (mean (95% CI) change 0–8 weeks 664 (473,854) cells/mm^3^) then gradually declined during the following 40 weeks (Figure 1b). In comparison, CD4 and CD8 cell counts in patients remaining on therapy (CT group) remained stable over the same 48 week period (CD4 and CD8 CT versus PTI global p<0.001) (Figure 1b).

### CD4 cell loss is due to reductions in both naïve and memory cells

**Figure 2:**
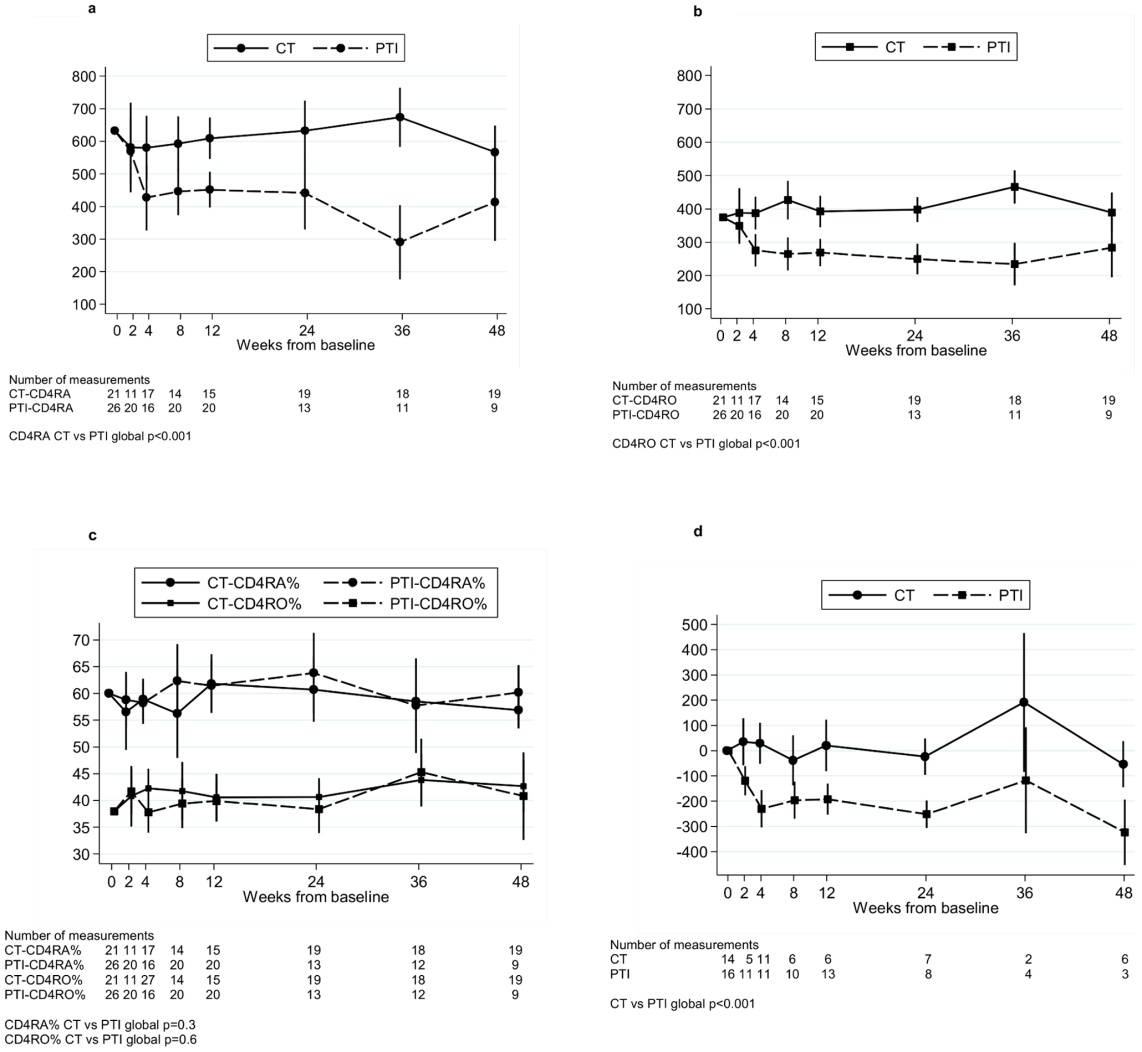
Changes in CD45RA, CD45RO and CD45A+CD31+ cells. Changes in absolute numbers of CD45RA cells (a), CD45RO cells (b) and CD4 RA and RO cells as a % of CD4 cells, for children in the trial where of CD4 sub-populations were evaluated (including the children in the substudy) (c). Change in CD45RA+ CD31+ cells from baseline for children in the substudy (d). CT  =  continuous ART (antiretroviral therapy), PTI  =  planned treatment interruption (off ART). Changes from baseline were estimated using normal regression of actual measurements adjusting for baseline using the overall mean at randomisation as the reference category.

The loss of CD4 cells following PTI was the result of a decrease in both naïve and memory cells; mean (95% CI) change 0–4 weeks was −204 (−305, −104) and −98 (−147, −50) cells/mm^3^ respectively (Figure 2a and 2b, data were available for ∼25–50% of children at each time point). Interestingly, as a consequence, the proportion of CD4 cells expressing CD45RA and CD45RO remained approximately constant throughout the first 48 weeks in both the CT and PTI (off ART) groups (Figure 2c, data were available for ∼25–50% of children at each time point, CD45RA% CT versus PTI global p = 0.3, CD45RO% CT versus PTI global p = 0.6). CD4 cells stained for CD31 and CD45RA (substudy children only) revealed four populations of cells (Figure S1). The largest population of cells (approximately 49%) were positive for both CD31 and CD45RA (possibly representing recent thymic emigrants cells). This subpopulation rapidly declined within the first 4 weeks off ART in the PTI group; mean (95% CI) change 0–4 weeks was −230 (−304, −156) versus +29 (−52,+110) cells/mm^3^ in CT group, p<0.001 (Figure 2d, global p<0.001). Reductions were seen in the other three cell populations, including the CD45RA- CD31- and CD45RA- CD31+ memory cells (data not shown).

### Increases in CD8 cells following PTI are due to increases in CD8 memory cells

**Figure 3:**
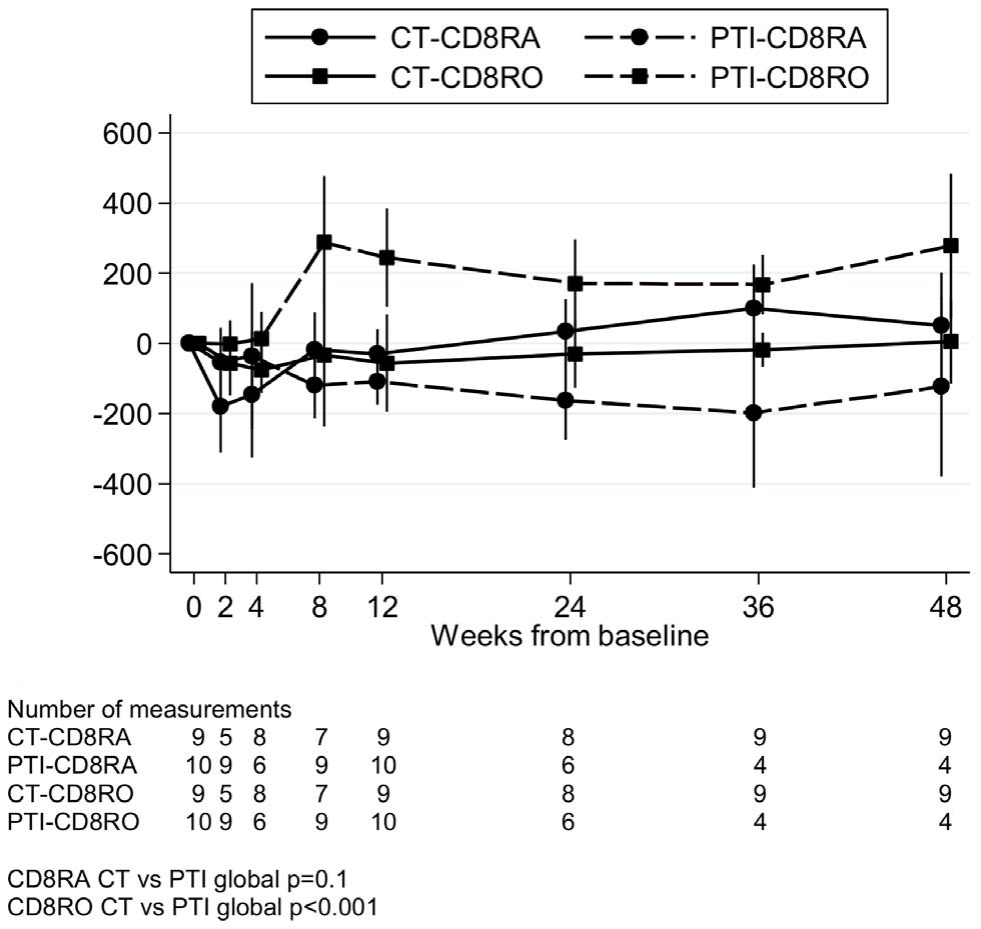
Change in CD8 RA and RO cells from baseline. Change in CD8 RA and RO cells from baseline for children in the substudy. CT  =  continuous ART (antiretroviral therapy), PTI  =  planned treatment interruption (off ART). Changes from baseline were estimated using normal regression of actual measurements adjusting for baseline using the overall mean at randomisation as the reference category.

Increases in CD8 cells following PTI seen in Figure 1b were predominantly due to increases in RO-expressing memory cells; mean (95% CI) change 0–8 weeks was +288 (+99,+476) versus −33 (−237,+171) cells/mm^3^ in CT (p = 0.04) (Figure 3, global p<0.001). This was concurrent with a small but persistent reduction in RA-expressing CD8 cells; mean 95% CI change 0–8 weeks was −119 (−213, −26) versus −19 (−125,+88) cells/mm^3^ in CT (p = 0.2). However, the ratio of RO to RA cells remained constant between 8 and 48 weeks off ART.

### PTI is associated with immune activation and proliferation

**Figure 4:**
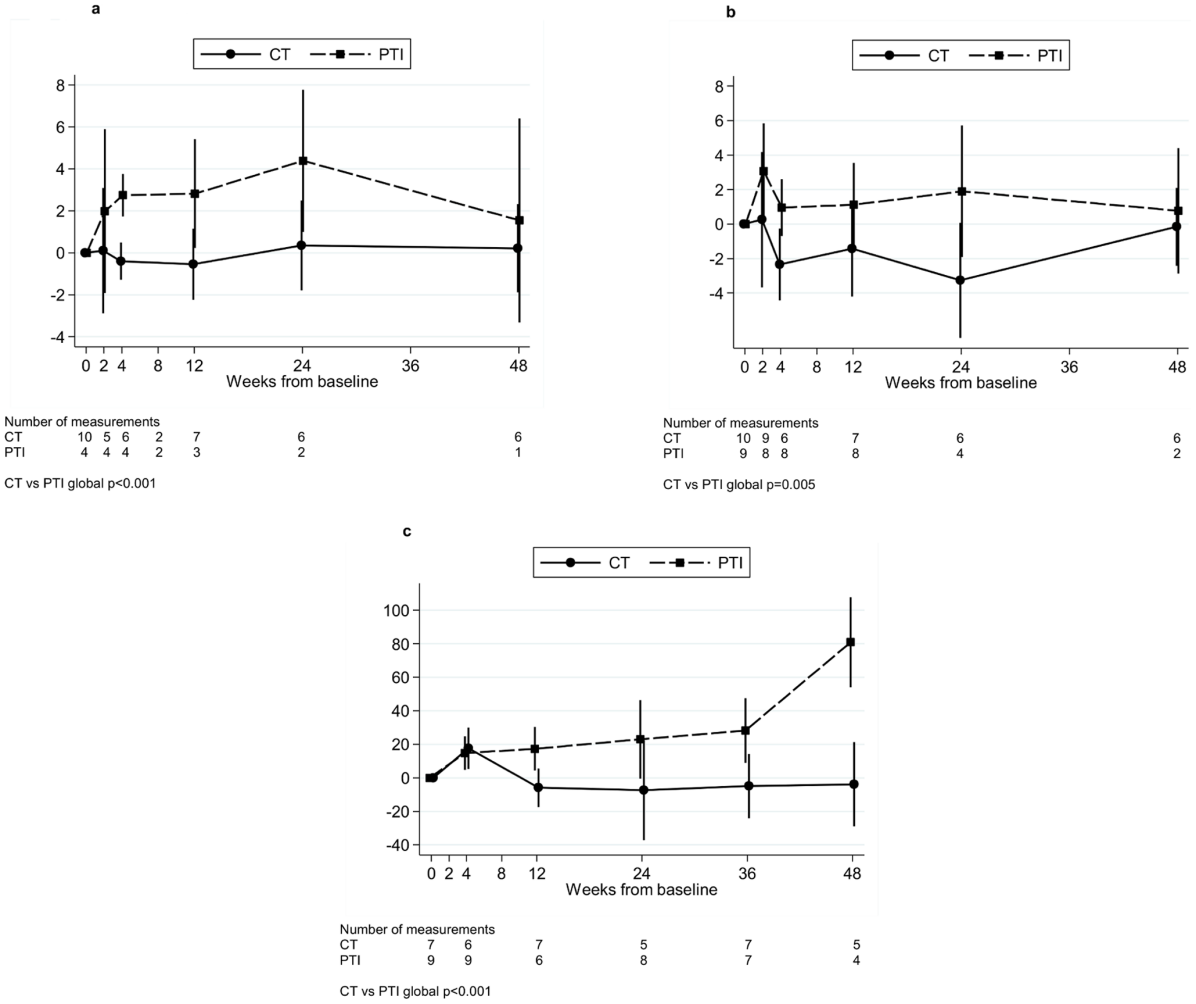
Change in % Ki67, HLA-DR and LPS from baseline. Change in % Ki67 (a) and % HLA-DR (b) in CD45RA- CD31- cells and in LPS (c) from baseline for children in the substudy. CT  =  continuous ART (antiretroviral therapy), PTI  =  planned treatment interruption (off ART). Changes from baseline were estimated using normal regression of actual measurements adjusting for baseline using the overall mean at randomisation as the reference category.

PTI was associated with a rapid increase in CD4 cells expressing Ki67 and HLA-DR 2 weeks after stopping ART, which was sustained subsequently. This was most marked in the CD4 CD45RA-CD31- (memory) population; mean (95% CI) change in percentage of cells expressing Ki67 0–4 weeks was 2.7% (1.7%,3.8%) in PTI versus −0.4% (−1.3%,+0.5%) CT, p = 0.004 (Figure 4a, global p<0.001); for HLA-DR, the mean (95% CI) change 0–4 weeks was 1.0% (−0.7%,+2.6%) PTI versus −2.3% (−4.4%,−0.3%) CT, p = 0.04, (Figure 4b, global p = 0.005). A similar trend was seen in the other subpopulations of CD4 cells, particularly CD45RA-CD31+ cells, which persisted throughout the 48 week period studied. There was also a trend towards increased expression of CD38 in the CD8 population in the PTI group, although numbers were small: mean (95% CI) change 0–4 weeks was +6.7% (−4.6%,+17.9%) in PTI (n = 3) versus +2.1% (−8.8%,+13.0%) CT (n = 5).

In the PTI group, mean (SD) LPS levels were 99 (25) pg/ml at baseline and increased by mean (95% CI) +15 (+5,+25) pg/ml by four weeks off ART versus +18 (+5,+30) CT, p = 0.7. Increases were sustained to 48 weeks, mean (95% CI) change 0–48 weeks was +81 (+54,+108) PTI versus −4 (−29,+21) pg/ml CT, p = 0.004. (Figure 4c, global p<0.001).

### PTI may be associated with increased HIV-1 DNA

**Figure 5:**
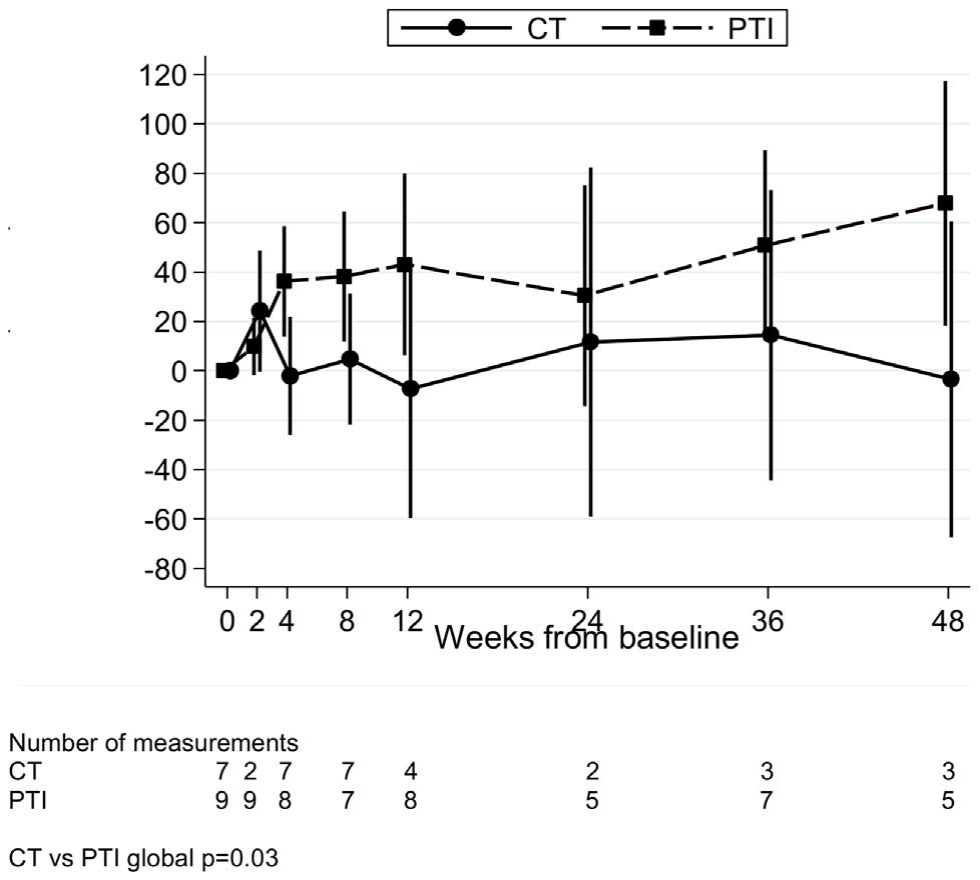
Relative change in HIV DNA copies/10^6^ PBMC from baseline. Relative change in HIV DNA copies/10^6^ PBMC from baseline for children in the substudy. CT  =  continuous ART (antiretroviral therapy), PTI  =  planned treatment interruption (off ART), PBMC  =  peripheral blood mononuclear cells.

By four weeks, levels of HIV DNA per 10^6^ PBMC had increased in the PTI group; mean (95% CI) relative change 0–4 weeks in log_10_ HIV DNA per 10^6^ PBMC was +36% (+14%,+59%) in PTI versus −2% (−26%,+22%) in CT, p = 0.04 (Figure 5, global p = 0.03). This trend continued over 48 weeks of follow-up, although small numbers of measurements at later time points make it difficult to draw reliable conclusions.

## Discussion

In this first pilot study to document the detailed immunological and virological responses to PTI in children with chronic HIV-1 infection, the main findings are that after stopping ART, there were rapid changes in viraemia and cell-associated virus, CD4 and CD8 cell numbers, affecting both naive and memory populations, and CD4 cell activation and proliferation. However, all of these parameters had stabilised by 12 weeks and remained similar for the remaining 48-week period off ART.

The substantial drop in CD4 cell count following PTI was expected and was broadly similar to the known response to treatment interruption, planned or otherwise in children and adults [16], [17]. It would appear, however, that there are subtle, but potentially important, differences in CD4 cell dynamics between children and adults following PTI. The studies of Thiebaut [17] and Davey [18] indicate that, in contrast to our study, adults appear to exhibit a more pronounced progressive decline in CD4 cells. This occurs in spite of increased CD4 cell turnover [18], indicating that in adults, this is insufficient to keep pace with the CD4 cell loss. In contrast, in children it would appear that increased cell turnover, as indicated by the elevated CD4 Ki67 expression, and possibly higher thymic output, is sufficient to maintain CD4 counts after the initial drop in CD4 cells following treatment cessation.

Unexpectedly, both naïve and memory cell numbers were depleted following treatment interruption, such that the proportion of CD4 cells expressing CD45RA and CD45RO remained constant throughout the first 48 weeks in both CT and PTI groups. It is difficult to assess how our observations compare with adults. The limited data that does exists in adults on relative changes in naïve/memory cell populations is based on short-term periods of structured treatment interruption (STI) in contrast to the longer period of 48 weeks used in this study. It would appear that in adults, throughout the course of periods of STI, the predominant populations affected are memory cells, with variable changes in the naïve populations [19], [20].

It has been established that in adults, changes in CD4 following ART initiation are predominantly due to redistribution of memory cells, followed later by an increase in the naïve cell population [21]. This biphasic reconstitution is less pronounced in children with the increase in CD4 cells emanating predominantly from the naïve CD4 pool [22]–[24]. Naïve CD4+ T cell numbers are maintained by a balance between production of naïve cells and their loss by death or differentiation. The chemokine IL-7 is a key homeostatic regulator of naïve T cell numbers, along with self-MHC stimulation [25]. The mechanisms by which memory cells are maintained are less well defined [25], but survival and self-renewing proliferation are likely to be controlled by competition for homeostatic resources such as cytokines, e.g. IL15, and TCR-self-peptide/MHC interactions [26]. In this study, the rapid decline in both naive and memory cells and re-stabilisation of both compartments at a new lower CD4 count implies that regardless of what may be driving the CD4 cell loss, a degree of homeostasis is achieved for both CD4 naïve and memory compartments, irrespective of the CD4 cell decline, which is then maintained for at least 48 weeks.

To investigate if PTI caused a selective loss of recent thymic emigrants, we stained CD4 cells with CD31, also known as platelet endothelial cell adhesion molecule-1. CD31+ CD45RA+ cells have a high T cell receptor excision circle (TREC) content, and are enriched with cells that have recently emigrated from the thymus. CD31–CD45RA+ cells represent a population of naïve cells with a lower TREC content in which CD31 expression is reduced in response to homeostatic expansion [27]. Two RA- populations defined by CD31 expression also exist. In healthy individuals the largest population is CD31-, with <5% expressing CD31. The latter population appears to have a lower replicative history than the CD31- cells [28]. It is possible to maintain the overall proportions of naïve and memory populations whilst seeing substantial shifts in the four populations of CD4 cells as defined by CD31 expression. For example, expansion of CD31- naïve cells may increase to compensate for a loss of recent thymic emigrants. Our data did not find evidence that this was the case and confirmed that the predominant populations affected by PTI were the CD31+CD45RA+ and CD31-CD45RA- cells.

The very rapid decline in CD4 cells combined with the observation that CD31+CD45RA+ cells were not the only population to diminish, would make a reduction in thymic output unlikely as the primary cause of the CD4 cell decline. A more plausible mechanism is immune activation, now a well recognised driver of CD4 cell death [29] which is also seen in adults following PTI [30], [31]. We observed a very rapid increase in both Ki67 and HLA-DR expression after PTI, which is indicative of increased cell turnover and CD4 cell activation. Both Ki67 and HLA-DR remained elevated during the period off ART, most marked in the memory populations. Taken together, our results suggest that while the CD4 count remained stable after 12 weeks of PTI, it was being maintained because of, or indeed in spite of, increased cell turnover. If the CD4 count after 12 weeks represents a new ‘CD4 set point’, it would thus appear to be maintained by an increase in cell proliferation, a concurrent increase in cell death and possibly a change in thymic output.

Immune activation has been described in adults not taking ART and has been linked to long-term adverse effects [32]. It is unclear if the level and duration of immune activation and increased CD4 cell turnover has long-term effects on the immune system and on other organs, such as the cardiovascular system in children. Two observations may be pertinent to this question. First, we observed increased levels of LPS following PTI. This could conceivably be because of CD4 cell depletion within the gastrointestinal tract, resulting in increased circulating microbial components, or modulation of intestinal flora [33], [34]. If increased LPS does indeed represent immune injury within the gastrointestinal tract, this may be irreversible. However evidence against irreversible immune injury comes from two observations. Firstly, that the proportion of RA to RO cells remained constant up to 48 weeks off ART. The absence of a selective decline in the naïve population may indicate that thymic output is being maintained at pre-PTI levels. In ongoing work in a Ugandan cohort, we have found that the proportion of CD45RA+CD31+ cells prior to starting therapy is the best indicator of eventual immune recovery [35], [36]. Secondly, two years after the end of the PENTA 11 trial, there were no statistically significant differences in CD4 cell counts between the continuous and treatment interrupted groups [2]. Detailed immunological and virological studies are ongoing and should provide further insights into the long-term effects of PTI in children, many of whom will interrupt ART at some stage in childhood, given the considerable challenges ART presents to families.

In contrast to CD4 cells, there was a rapid rise in CD8 memory cells after ART interruption. The most plausible explanation is that this represents expansion of CD8 clones directed against HIV [37]. It is also possible that some of the increase is due to proliferation of CD8 cells directed against non-HIV epitopes, such as herpes viruses, or as a result of bystander activation. The sustained increase in CD8 cells indicates that the drivers of the CD8 expansion were persistent, and the observed increase in CD8+CD38+ expression with PTI would be consistent with increased immune activation [38]. A lack of CD8 effectiveness in controlling HIV may be reflected in the increase in HIV-1 DNA observed as early as 4 weeks and persisting for the duration of PTI. It will be important to ascertain if this apparent increase in HIV-1 DNA persists even after ART reintroduction, which we are now studying.

A limitation of this study was the small patient population sampled. This was due to both low numbers enrolled in the sub study, and technical failures, namely inadequate numbers of cells or poor recovery following thawing. A further limitation was only having the capacity at the time, to analyse four colours by flow cytometry. This restricted the range of markers that could be analysed, such as CD27 and CCR7 to further characterise the naïve and memory populations.

Overall, PTI in children results in rapid changes in CD4 and CD8 cells, which are associated with increased cell turnover and immune activation. The CD4 cell decline appears to be due to changes in both naïve and memory cell populations, such that their ratio remains stable throughout the period of treatment interruption. Taken together, these results suggest that, in the face of ongoing immune activation as a result of PTI, these children are able to maintain their circulating naïve CD4 cells, at least in proportion to their circulating memory cell pool. This may indicate that PTI of up to 48 weeks in children during mid-childhood who have been on ART for >5 years, is not necessarily associated with irreversable degradation of the naïve cell pool. This may explain the degree of CD4 cell recovery which we have observed following ART re-introduction, whereby CD4 counts and % among children in the PTI group ‘caught up’ with children on CT by 2 years back on ART [2]. In the light of our findings, it is interesting that children who received multiple cycles of PTI, followed by ART, showed evidence of enhanced immunity to HIV, and lower plasma RNA during episodes of treatment interruption. This may indicate that there may even be some potential benefits from controlled exposure to viral antigens [39]. Further studies are required to fully evaluate the long-term consequences of PTI in children before the benefits and risks can be objectively assessed. However, while PTI is not recommended, it could be considered when the risk of unplanned treatment interruptions is high.

## Conclusion

This is the first study to document in detail the impact of treatment interruption on CD4 and CD8 dynamics in the context of a randomised clinical trial. The data presented indicate that changes occur within weeks, and include a reduction in naïve and memory CD4 cell populations and an increase in CD8 memory cells. These changes persist for the remainder of the 48 weeks following treatment interruption. These changes were associated with an increase in CD4 and CD8 activation and proliferation, which persisted for the duration of the period of treatment interruption. By the end of 48 weeks the ratio of naïve to memory cells was similar to that found at baseline and may indicate that changes in T cell dynamics were not necessarily irreversible. This would be consistent with the excellent long-term recovery of CD4 cells following re-initiation of treatment.

## Supporting Information

Figure S1 **Representative FACS Profiles.** Dot Plot: CD4 cells stained for CD31 and CD45RA revealed four populations of cells. The largest population of cells were positive for both CD31 and CD45RA. Representative Histograms are shown for Ki67 and HLA-DR for CT  =  continuous ART (antiretroviral therapy), and PTI  =  planned treatment interruption (off ART).
(TIFF)
