# Supplementary figures and images for: The Immunological and Virological Consequences of Planned Treatment Interruptions in Children with HIV Infection
Source: PLoS One. 2013 Oct 23;8(10):e76582. doi: 10.1371/journal.pone.0076582 (PMC3806774; doi:10.1371/journal.pone.0076582)

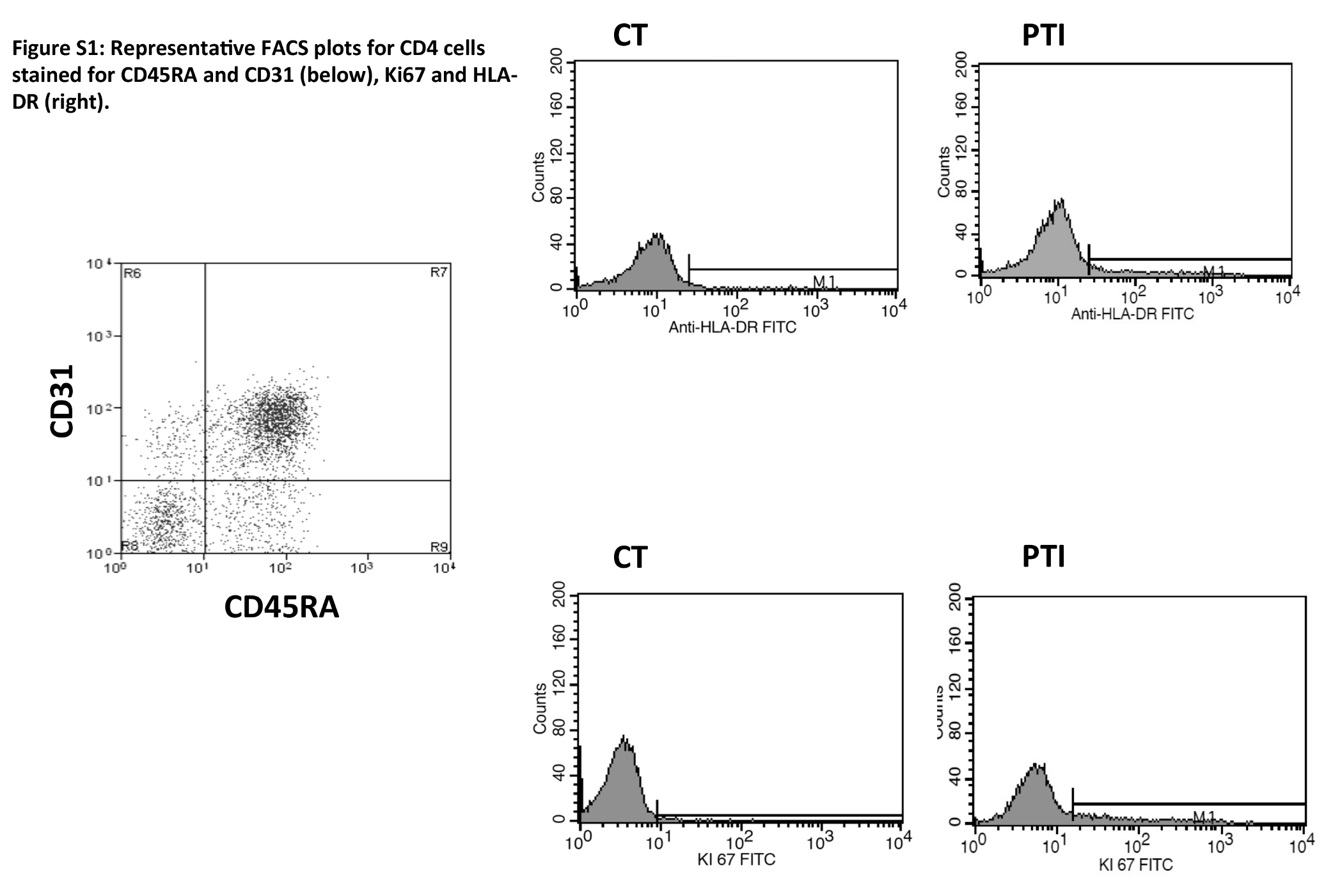

Supplement: Figure S1 — Representative FACS Profiles. Dot Plot: CD4 cells stained for CD31 and CD45RA revealed four populations of cells. The largest population of cells were positive for both CD31 and CD45RA. Representative Histograms are shown for Ki67 and HLA-DR for CT = continuous ART (antiretroviral therapy), and PTI = planned treatment interruption (off ART). (TIFF) [file pone.0076582.s001.tiff]
